# Craniosynostosis affects the majority of mucopolysaccharidosis patients and can contribute to increased intracranial pressure

**DOI:** 10.1007/s10545-018-0212-1

**Published:** 2018-08-06

**Authors:** Esmee Oussoren, Irene M. J. Mathijssen, Margreet Wagenmakers, Rob M. Verdijk, Hansje H. Bredero-Boelhouwer, Marie-Lise C. van Veelen-Vincent, Jan C. van der Meijden, Johanna M. P. van den Hout, George J. G. Ruijter, Ans T. van der Ploeg, Mirjam Langeveld

**Affiliations:** 1 Department of Pediatrics, Center for Lysosomal and Metabolic Diseases, Erasmus MC—Sophia Children’s Hospital, P.O. Box 2060, 3000 CB Rotterdam, The Netherlands; 2 Department of Plastic, Reconstructive and Hand Surgery, Dutch Craniofacial Centre, Erasmus MC, Rotterdam, The Netherlands; 3 Department of Internal Medicine, Center for Lysosomal and Metabolic Diseases, Erasmus MC, Rotterdam, The Netherlands; 4 Department of Pathology, Erasmus MC, Rotterdam, The Netherlands; 5 Department of Neurosurgery, Erasmus MC, Rotterdam, The Netherlands; 6 Department of Clinical Genetics, Center for Lysosomal and Metabolic Diseases, Erasmus MC, Rotterdam, The Netherlands

## Abstract

**Background:**

The mucopolysaccharidoses are multisystem lysosomal storage diseases characterized by extensive skeletal deformities, including skull abnormalities. The objective of this study was to determine the incidence of craniosynostosis in the different mucopolysaccharidosis (MPS) types and its clinical consequences.

**Methods:**

In a prospective cohort study spanning 10 years, skull imaging and clinical evaluations were performed in 47 MPS patients (type I, II, VI, and VII). A total of 215 radiographs of the skull were analyzed. The presence and type of craniosynostosis, the sutures involved, progression over time, skull shape, head circumference, fundoscopy, and ventriculoperitoneal shunt (VPS) placement data were evaluated.

**Results:**

Craniosynostosis of at least one suture was present in 77% of all 47 MPS patients (≤ 6 years of age in 40% of all patients). In 32% of all MPS patients, premature closure of all sutures was seen (≤ 6 years of age in 13% of all patients). All patients with early closure had a more severe MPS phenotype, both in the neuronopathic (MPS I, II) and non-neuronopathic (MPS VI) patient groups. Because of symptomatic increased intracranial pressure (ICP), a VPS was placed in six patients, with craniosynostosis as a likely or certain causative factor for the increased pressure in four patients. One patient underwent cranial vault expansion because of severe craniosynostosis.

**Conclusions:**

Craniosynostosis occurs in the majority of MPS patients. Since the clinical consequences can be severe and surgical intervention is possible, skull growth and signs and symptoms of increased ICP should be monitored in both neuronopathic and non-neuronopathic patients with MPS.

**Electronic supplementary material:**

The online version of this article (10.1007/s10545-018-0212-1) contains supplementary material, which is available to authorized users.

## Introduction

Mucopolysaccharidoses are lysosomal storage diseases caused by deficiencies of glycosaminoglycan (GAG)-degrading enzymes. The mucopolysaccharidoses are multisystem disorders with a broad range of clinical manifestations, including extensive skeletal abnormalities (dysostosis multiplex, joint contractures) and hydrocephalus (Neufeld and Muenzer 2001; Dalla Corte et al. 2017). Neurological decline due to GAG accumulation in the brain is seen in a subset of patients with mucopolysaccharidosis (MPS) type I, II, III, and VII (Neufeld and Muenzer 2001; Shapiro et al. 2017).

In healthy individuals, the skull expands by growth from the sutures up to the age of 6 years. After the age of 6 years, both sutural and appositional growth takes place (Cohen 1988, 1993). The metopic suture closes between the age of 3 and 9 months (Vu et al. 2001). The sagittal, coronal, and lambdoid sutures begin to close much later, around, respectively, 22, 24, and 26 years of age (Cohen 1993). If one or more suture(s) close(s) at an earlier age, this can result in growth stagnation and/or an abnormal skull shape. This premature fusion (craniosynostosis) can be classified as simple (one fused suture) or complex (multiple sutures involved), primary (sutural biology abnormality) or secondary (due to external influences), and as part of a syndrome or isolated (Moosa and Wollnik 2016).

Early closure of each suture results in a different shape of the skull; for example, early closure of the sagittal suture results in an elongated and narrow skull (scaphocephaly) and early closure of the lambdoid sutures results in occipital flattening (pachycephaly) (Persing et al. 1989). Early-onset craniosynostosis, defined as closure of sutures before the age of 6 years, can restrict skull growth and can cause elevated intracranial pressure (ICP), which in turn can result in visual impairment (de Jong et al 2010). Therefore early recognition of craniosynostosis is of great importance. Timely surgical intervention can provide space for the brain to grow, preserving development and vision (Speltz et al 2004)

Up till now, only two small cross-sectional studies investigated the prevalence of craniosynostosis in MPS patients. They found secondary craniosynostosis in 19% (7 out of 36) of severe MPS II patients and 11% (2 out of 18) of MPS IVA patients. In addition, three case reports describe the presence of craniosynostosis in MPS (types I and VI) (Taylor et al. 1978; Cohen 1993; Brisman et al. 2004; Manara et al. 2011; Ziyadeh et al. 2013; Bhattacharya et al. 2014; Sadashiva et al. 2015). The incidence and type of craniosynostosis in MPS, the development over time, severity, and clinical consequences have not been studied. In our prospective study, this was systematically evaluated in a relatively large cohort of patients with MPS I, II, VI, and VII.

## Methods

### Patients

From 2007 onwards, all pediatric patients with MPS (type I, II, VI, and VII) treated at the Center for Lysosomal and Metabolic Diseases of the Erasmus MC, Rotterdam, the Netherlands, were included in a prospective cohort study. In all patients, the diagnosis of MPS had been confirmed by the measurement of enzyme deficiency in leukocytes or fibroblasts and by DNA analysis. Yearly evaluation was done according to a standardized follow-up protocol, which included medical history taking, physical and neurological examination, skull radiographs, and ophthalmological examinations. Available data from 2002 till 2007 were added retrospectively. The study was approved by the Medical Ethical Review board at the Erasmus MC.

### Radiographic evaluation of sutures and skull shape

Skull radiographs [anterior posterior (AP) and lateral] were obtained yearly up to the age of 18 years. Radiographs of patients with a ventriculoperitoneal shunt (VPS) were excluded after drain placement, as the drain itself can induce secondary closure of one or more sutures in proximity to the drain (Ryoo et al. 2014). Furthermore, the postoperative radiographs of a patient who underwent cranial surgery were excluded from the analysis.

Each radiograph was evaluated by two independent observers (craniosynostosis expert and plastic surgeon professor IMJM and lysosomal expert and metabolic pediatrician EO). In each radiograph, three sutures (coronal, sagittal, and lambdoid) were scored as open, partially closed, or closed. The metopic suture was not analyzed as it physiologically closes between the age of 3 and 9 months and, for most patients, radiographs were not available at such an early age (Vu et al. 2001).

For each MPS type (I, II, VI, and VII), the proportion of patients with craniosynostosis was determined. Furthermore, the proportion of patients with one closed suture and with two or more closed sutures was determined, and the order in which the sutures closed was analyzed. For each patient, the shape of the skull was described using the last available radiograph. Of three patients with craniosynostosis, three-dimensional computed tomography (CT) scans of the skull were available.

### Head circumferences, ophthalmological and physical examination

Head circumference was measured at least yearly and the measurements closest to the evaluated radiographs of the skull were used for analysis. At physical examination, head shape and facial features were examined.

The presence of raised ICP was evaluated by fundoscopy during yearly ophthalmological assessments, unless fundoscopy could not be reliably performed due to corneal clouding or behavioral problems.

### VPS placement

Of the MPS patients who received a VPS, the following parameters at the time of placement were determined: age, VPS indication [clinical features, brain imaging, and cerebrospinal fluid pressure (CSF)], head circumference, head shape, presence or absence of craniosynostosis, and result of fundoscopy.

### Statistics

All data are presented as median and range, unless otherwise stated.

## Results

### Patient data and characteristics

Forty-seven patients with MPS (type I, II, VI, and VII) were included in this study (72% male patients). The median age at diagnosis was 2.8 (range 0–12) years. Table 1 shows the disease type and severity, mutation(s), gender, age at diagnosis, age at first and last radiograph, and skull shape for each patient.

**Table 1:** Patient characteristics

MPS I	Hurler (H)/Hurler/Scheie (HS)/Scheie (S)	*IDUA* gene/protein	Gender, male (M)/female (F)	Age at diagnosis (years)	Age first X skull (years)	Age last X skull (years)	Skull shape last radiograph
1	H	p.Q70X/p.L218P	M	2.4	11.8		Normal
2	H	p.Q70X/p.W402X	F	2	6.1		Scaphocephaly, pachycephaly
3	H	p.Q70X/p.L218P	M	1.7	6.2	8.1	Pachycephaly
4	H	p.Q70X/p.L218P	M	0.9	1		Scaphocephaly
5	H	p.Q70X/p.A327P	M	1	0.8		Pachycephaly
6#	H	p.Q70X/p.Q70X	F	1	1.2	2.2	Scaphocephaly
7	H	p.Q70X/p.L218P	M	0.9	1.0	3.2	Pachycephaly
8	H	p.A327P/p.A327P	F	1.3	1		Scaphocephaly
9	H	c.1273dup, p.H425fs/c.1273dup, p.H425fs	F	0.7	2.7		Scaphocephaly
10#	H	p.Q70X/Q70X	M	0	0	4.6	Normal
11	H	c.1893del, p.F632 fs/c.1893del, p.F632 fs	F	1.2	1.2		Scaphocephaly
12	H/S	p.W402X/p.W402X	M	1	0.4		Pachycephaly
13	S	p.W402X/n.i.	M	5	7.9	14.5	Normal
14	S	p.R383H/c.474-2A>G	F	2.3	2.6		Normal
MPS II	Neuronopathic (N)/non-neuronopathic (NN)	*IDS* gene/protein	Gender, male (M)/female (F)	Age at diagnosis (years)	Age first X skull (years)	Age last X skull (years)	Skull shape last radiograph
1	N	p.S349R	M	3	10.3	15.3	Normal
2	N	p.E521K	M	3	11.2		Pachycephaly
3	N	p.P86L	M	6	9.7		Pachycephaly
4	N	p.E459*	M	2	8.7	13.7	Normal
5	N	p.S333 L	M	3	3.1		Normal
6	N	p.S117del	M	0	0.2		Normal
7	N	c.1511del, p.G504 fs	M	4.7	5.5	8.6	Normal
8	N	c.544del, p.L182 fs	M	2	2.4		Normal
9	N	Total IDS del^	M	4	7.1	7.7	Normal
10	N	p.S333 L	M	2	0.2	1.7 (CT scan)	Plagiocephaly anterior right
11	N	p.L522P	M	1	1.1		Normal
12	Unknown	p.H229R	M	5	6.7		Normal
13$	NN	p.F137S	M	3.3	3.5	6.9	Scaphocephaly
14$	NN	p.F137S	M	3.3	3.5	7.2	Scaphocephaly
15	NN	p.Y225D	M	4.1	4.2	5.7	Pachycephaly
MPS VI	Rapidly progressive (R)/slowly progressive (S)	*ARSB* gene/protein	Gender, male (M)/female (F)	Age at diagnosis (years)	Age first X skull (years)	Age last X skull (years)	Skull shape last radiograph
1	R	c.1142+2T>C, p?/c.1142+2T>C, p?	F	2.9	5.7	14.8	Normal
2	R	p.P313S/p.P313S	M	12	12	13	Scaphocephaly
3	R	p.V332G/p.V332G	M	2.7	2.9		Pachycephaly
4	R	p.P313S/p.P313S	F	6.5	8.6		Scaphocephaly
5	R	p.N301 K/p.N301 K	F	1.9	2.5	8.6	Normal
6	R	p.G324 V/p.G324 V	M	1.4	2.2	8.2	Scaphocephaly
7£	R	p.P313A/p.P313A	F	4.6	4.6	6.6	Normal
8	R	Unknown	F	3.1	3.1	4.5 (CT scan)	Scaphocephaly
9£	R	p.P313A/p.P313A	M	2.2	2.4	4.4	Normal
10	R	p.H141P/p.L321P	M	1.9	2.0	2.1 (CT scan)	Pachycephaly, brachycephaly
11	S	p.R152W/p.R152W	F	7.5	7.8	14	Normal
12	S	p.Y210C/p.P313A	M	10.3	12.4	14.4	Normal
13	S	p.R152W/p.R152W	M	0.7	7.7	15.1	Normal
14&	S	p.Y210C/p.R327X	F	6.4	7.3	15.3	Normal
15&	S	p.Y210C/p.R327X	M	5	5.3	14.3	Normal
16	S	p.Y210C/p.R327X	M	5.9	5.8	13.1	Normal
MPS VII	Mild/severe	*GUSB* gene/protein	Gender, male (M)/female (F)	Age at diagnosis (years)	Age first X skull (years)	Age last X skull (years)	Skull shape last radiograph
1α	Mild	p.V99 M/p.V99 M	M	8.4	8.4		Normal
2α	Mild	p.V99 M/p.V99 M	M	6.7	6.7		Pachycephaly

#, $, £, &, and α: siblings

The patient numbering in the first column is the same as in Fig. 2

### Skull radiographs

A total of 215 skull radiographs were analyzed. The first available radiograph was taken at 4.2 (range 0–12.4) years of age for the entire MPS patient group. The follow-up period was 3.4 (range 0.1–9.1) years (Table 1).

### Prevalence of craniosynostosis in MPS

**Fig. 1:**
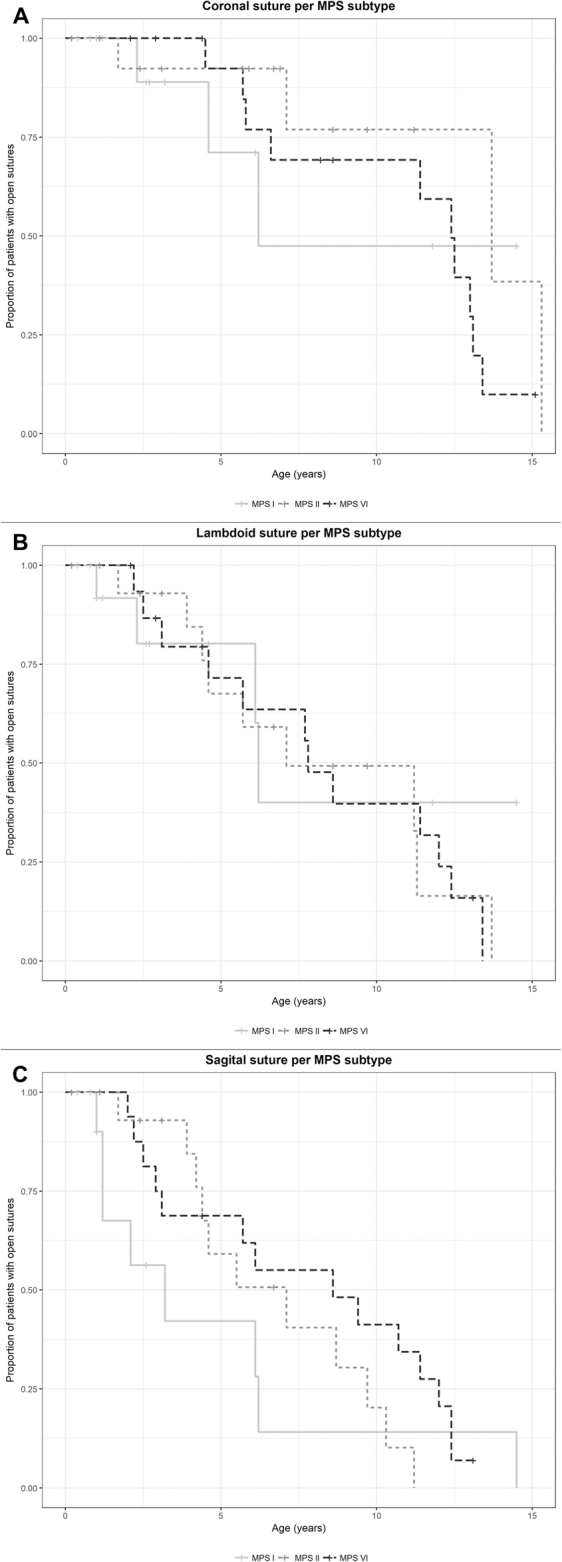
Suture closure in the different mucopolysaccharidosis (MPS) types. Kaplan–Meier curves of open suture(s) (coronal: A, lambdoid: B, and sagittal: C) by MPS subtype (types I, II, and VI ). 1.0 means 100% of patients with open or partially closed suture, 0 means suture closed in all patients. MPS VII patients were not included in the graph because of the low number (n = 2). In healthy individuals, the sagittal, coronal, and lambdoid sutures begin to close around, respectively, 22, 24, and 26 years of age

**Fig. 2:**
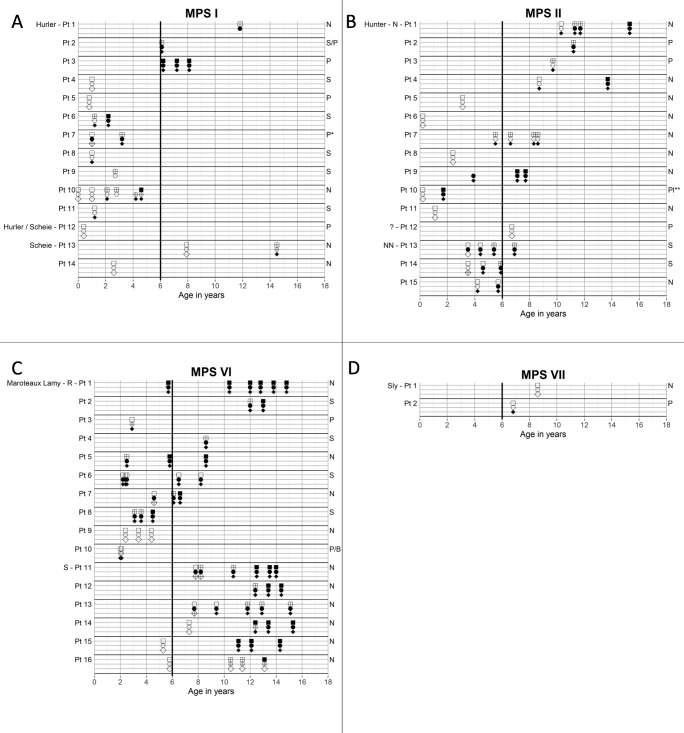
Craniosynostosis by MPS type in individual patients. For each MPS type, the suture closure over time is shown. Each suture is depicted by a symbol: coronal: square; lambdoid: circle; sagittal: diamond. Closure status is indicated by the filling of the symbol: transparent: open suture(s); shaded: partial closure; black: completely closed. For MPS I, II, and VI, the most severe phenotypes are at the top and the least severe phenotypes are at the bottom of the graph. Abbreviations in the graph: Pt.: patient; N: neuronopathic; NN: non-neuronopathic R: rapidly progressive; S: slowly progressive. Right: skull shape; N: normocephaly; S: scaphocephaly; P: pachycephaly; B: brachycephaly; Pl*: plagiocephaly. A black line is drawn at the age of 6 years; if suture closure occurs before the age of 6 years, this is regarded as early-onset craniosynostosis

Craniosynostosis of at least one suture was present in 77% of all 47 MPS patients and in 40% of patients, this occurred before the age of 6 years (Figs. 1 and 2). Table 2 shows the prevalence of craniosynostosis, the number of sutures involved, and the resulting head shape for each MPS type.

**Table 2:** Prevalence of craniosynostosis, the number of sutures involved, and the resulting skull shape by mucopolysaccharidosis (MPS) type

	Total MPS	MPS I	MPS II	MPS VI	MPS VII
≥ 1 Suture closed; *n*/total (%)	36/47 (77%)	10/14 (71%)	10/15 (67%)	15/16 (94%)	1/2 (50%)
≥ 1 Suture closed ≤ 6 years old; *n*/total (%)	19/47 (40%)	6/14 (43%)	6/15 (40%)	7/16 (44%)	0/2 (0%)
1 Suture closed; *n*/total (%)	4/47 (9%)	2/14 (14%)	0/15 (0%)	1/16 (6%)	1/2 (50%)
> 2 Sutures closed; *n*/total (%)	31/47 (66%)	7/14 (50%)	10/15 (67%)	14/16 (88%)	0/2 (0%)
All sutures closed; *n*/total (%)	15/47 (32%)	2/14 (14%)	4/15 (27%)	9/16 (56%)	0/2 (0%)
All sutures closed ≤ 6 years old; *n*/total (%)	6/47 (13%)	2/14 (13%)	1/15 (7%)	3/16 (19%)	0/2 (0%)
All sutures open; *n*/total (%)	11/47 (23%)	4/14 (29%)	5/15 (33%)	1/16 (6%)	1/2 (50%)
Skull shape; *n*/total (%)*	N: 24/47 (51%)	N: 4/14 (29%)	N: 9/15 (60%)^	N: 10/16 (63%)	N: 1/2 (50%)
	S: 12/47 (26%)	S: 6/14 (43%)#	S: 2/15 (13%)^	S: 4/16 (25%)	S: 0/2 (0%)
	P: 11/47 (23%)	P: 5/14 (36%)#	P: 3/15 (20%)^	P: 2/16 (13%)	P: 1/2 (50%)

*N: Normocephalic; S: scaphocephalic; P: pachycephalic

#One patient had both scaphocephalic and pachycephalic head shape

^One patient had only plagiocephaly at the right anterior side of the head and, therefore, the data of one patient are missing

### Sutures involved, progression over time, and severity

Of all MPS patients, 9% had premature (partial) closure of only one suture. In 66% of all MPS patients, two or three sutures partially or fully closed prematurely (Fig. 1 and Table 2). The longitudinal data on closure of each suture in each individual MPS patient are depicted in Fig. 2. In most cases, two or three sutures were already (partially) closed at the time the first radiograph was made (Fig. 2). The coronal suture was never the first to close (Fig. 1).

In 32% of all MPS patients, premature closure of all sutures was seen (≤ 6 years of age in 13% of all patients; Table 2). All these patients with early closure (≤ 6 years of age) had a more severe MPS phenotype.

### Skull shape

No specific skull shape abnormality (normocephaly) could be detected in 51% of all MPS patients (Tables 1 and 2). Despite a normal skull shape, all sutures had closed before the age of 6 years in two patients (MPS VI, patient nos. 1 and 5); this is also referred to as pansynostosis (Foo et al. 2010).

**Fig. 3:**
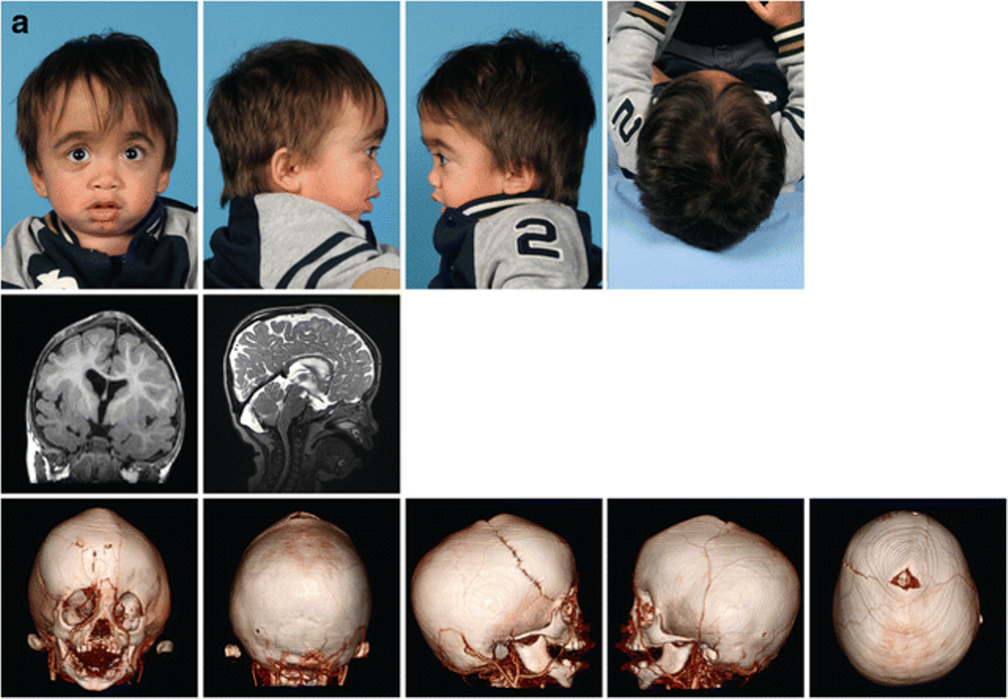

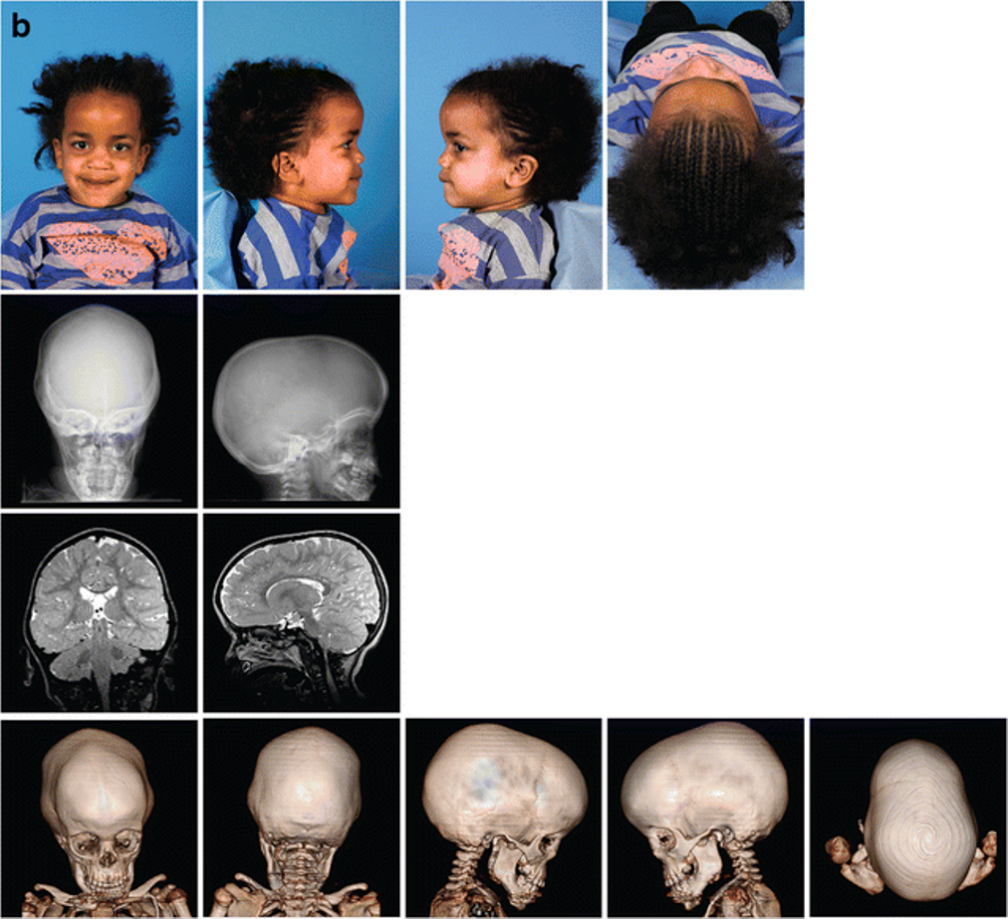

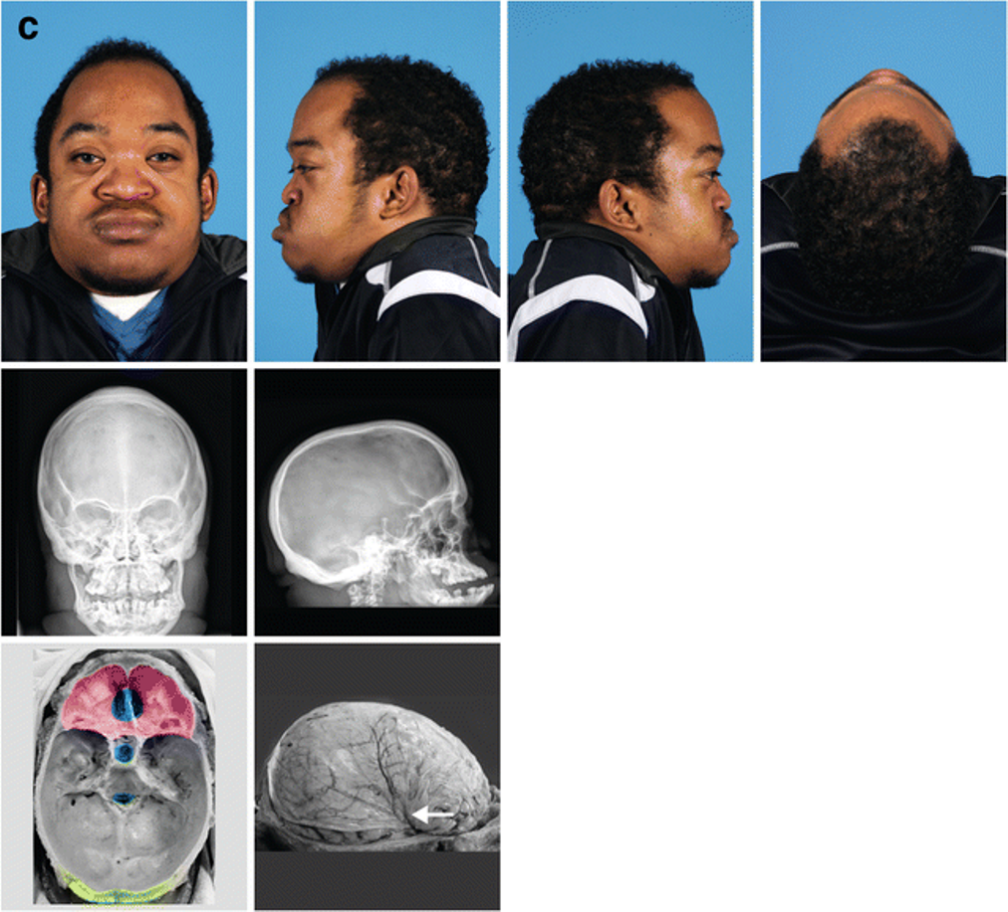
Three illustrative cases of craniosynostosis in MPS. **a** Patient no. 10, MPS II, 1.5 years old. Photographs show the distinct facial features, MRI T2 FLAIR and 3D CT scan shows the bulging anterior fontanel (volcano sign) with plagiocephaly at the right anterior side of the head. 3D CT scan shows the premature closure of the left coronal suture, the sagittal suture, and both lambdoid sutures. **b** Patient no. 8, MPS VI, 3.3 years old. Skull X-ray shows frontal bossing and scaphocephaly. X-skull and 3D CT scan show craniosynostosis of the metopic, both lambdoid and sagittal sutures. On MRI T2 FLAIR of the cerebrum, there are no signs of increased intracranial pressure (ICP). c MPS VI patient, 24 years old. Photographs and skull X-rays show distinct facial features, scaphocephaly, and sinus transversus impression in the skull. Macroscopic pictures from the autopsy at age 25 years show (in red) impressions of the brain gyri in the frontal bones and abnormal thin bone layer. In blue (top to bottom), abnormal deep olfactory furrow, sella turcica, and severe narrow foramen magnum. In green, thickened skull. The white arrow shows where there is an impression of the skull in the brain

Scaphocephaly was seen in 26% of all MPS patients. In 8 of these 12 patients, closure of the sagittal suture had occurred around or before the age of 6 years. Pachycephaly was detected in 23% of all MPS patients. Pachycephaly with scaphocephaly was seen in one patient (MPS I, patient no. 2). Plagiocephaly at the right anterior side of the head was seen in one MPS II patient (Fig. 3a, patient no. 10).

### Illustrative cases of craniosynostosis in MPS

Figure 3 shows photographs, skull X-rays, 3D CT scans, and MRI of the cerebrum of an MPS II patient (patient no. 10) (Fig. 3a) with bulging anterior fontanel due to craniosynostosis, an MPS VI patient (patient no. 8) (Fig. 3b) with frontal bossing and scaphocephaly due to craniosynostosis, and an adult MPS VI patient (brother of patient no. 4) (Fig. 3c). This adult patient died unexpectedly at 25 years of age as a result of a respiratory tract infection in an already highly compromised respiratory setting. Figure 3c shows macroscopic pictures from the brain autopsy with impression of the sinus transversus in the skull (X-skull) and impression of the brain gyri in the frontal bone, resulting from raised ICP earlier in life.

### Head circumferences

Data from at least one head circumferences measurement were available for 94% of all MPS patients. Only two patients had a head circumference outside the normal reference range (− 2SD). Both were MPS VI patients (patient nos. 1 and 5) in whom all sutures closed before the age of 6 years with stagnation (arrest) of skull growth. After the age of 6 years, their head circumferences started to increase again, in line with appositional growth of the skull. Supplemental Fig. 1 shows skull and height growth curves and photo- and radiographs of one of these patients (MPS VI, patient no. 1).

### Ophthalmic examination

Fundoscopy data were available for 85% of all MPS patients. Two patients had papilledema. In one of them, craniosynostosis was recognized at that time as the cause of increased ICP. Occipital cranial vault expansion with fronto-supraorbital remodulation surgery was performed at the age of 1 year and 9 months (MPS II, patient no. 10, Fig. 3a). In the second patient (MPS VI, patient no. 7), edema was mild, there were no clinical signs of increased ICP, and skull growth was normal; thus, an intervention was deemed unnecessary.

### VPS placement

In six patients, a VPS was placed at a median age of 5.4 (range 2–7.5) years, because of suspected elevated ICP (Table 3). Fundoscopy was performed or attempted in three patients before VPS placement. Two patients had no papilledema and in the other patient, fundoscopy was not possible due to corneal clouding or abnormal behavior. In five of the six patients, CT or MRI scan of the brain prior to VPS placement showed progressive hydrocephalus. They had clinical symptoms caused by the increased ICP, such as neurological decline (faster than the expected decline due to GAG accumulation in the brain), headache, and epilepsy. In four patients, the SD value of the head circumferences increased before VPS placement. In one patient (MPS II, patient no. 6), this occurred in a relatively short time (increase from 1 to 4SD), with only slightly elevated CSF pressure measured at the time of VPS placement. In one patient (MPS VI, patient no. 3), the head circumferences SD value declined prior to drain placement. This patient had partial closure of the lambdoid suture and complete closure of the sagittal suture with elevated CSF pressure (30–45 cm H2O) at the time of VPS placement (Fig. 2). We concluded that craniosynostosis, in combination with hydrocephalus, most likely contributed to the increased ICP in four out of the six patients with a VPS (Table 3; MPS I, patient nos. 2, 4, and 6; MPS VI, patient no. 3). In the fifth patient, craniosynostosis was not present (MPS II, patient no. 5) and in the sixth patient (MPS II, patient no. 6), no radiograph in the 2 years before shunt placement was made. One year after VPS placement, all sutures were closed in this patient (Fig. 2), suggesting this had already, at least partially, occurred before drain placement.

**Table 3:** Ventriculoperitoneal shunt (VPS) placement results by MPS type

	MPS I (patient no. 2)	MPS I (patient no. 4)	MPS I (patient no. 6)	MPS II (patient no. 5)	MPS II (patient no. 6)	MPS VI (patient no. 3)
Age at VPS placement (years)	7.5	2	5.3	7.5	2.5	5.5
Reason for VPS placement						
- Clinical	Neurological decline	Neurological decline; headaches	Reduced concentration	Neurological decline; epilepsy	Neurological decline	Headaches
- Brain imaging	CT: mild progression triventricular hydrocephalus	CT: progression hydrocephalus, bulging fontanel	MRI: progression hydrocephalus	MRI: hydrocephalus	MRI: mild progression quadriventricular hydrocephalus	MRI: no signs hydrocephalus
- CSF pressure*	NP	NP	NP	NP	20 mmHg	30–45 cm H2O
Head circumferences in SD values at the time of VPS placement	+ 1.5SD	+ 2.5SD	+ 2SD	+ 2.5SD	+ 4SD	− 1SD
Head shape^$^	S/P	S	S	N	N	S
Craniosynostosis present before VPS placement	Yes	Highly susceptible (bulging fontanel)	Yes	No	NP	Yes
Papilledema (fundoscopy)	No	NP	Not possible: corneal clouding	NP	NP	No

NP: Not performed; SD: standard deviation; CSF: cerebrospinal fluid

*CSF pressure of > 25 to 30 cm H2O (18–22 mmHg) is considered an indication for VPS

^$^S: Scaphocephaly; P: pachycephaly; N: normocephaly

## Discussion

This is the first long-term prospective study assessing skull suture closure and its consequences in patients with MPS I, II, VI, and VII. Our results show that craniosynostosis occurs at a very high frequency in these different types of MPS. Premature closure of at least one suture was present in 77% of patients, with suture closure before the age of 6 years in 40% of patients. In the general population, non-syndromic craniosynostosis occurs with a frequency between 0.4 and 1.0 per 1000 live births, while syndromic craniosynostosis is even more rare (Shuper et al. 1985; French et al. 1990; Singer et al. 1999; Boulet et al. 2008; Tahiri et al. 2017). The incidence found in the current study may even be an underestimation, because only one skull radiograph was available for 43% of our patients. In addition, the median follow-up was relatively short (3.4 years).

Consistent with syndromic craniosynostosis, the majority of MPS patients (66%) had early closure of more than one suture, with involvement of the lambdoid and coronal sutures (Twigg and Wilkie 2015).

An abnormal head shape resulting from early suture closure was seen in about half of all studied MPS patients, and scaphocephaly and pachycephaly were the most frequently observed. The trichonocephalic head shape which is seen by early closure of the metopic suture was not present in our MPS population.

Scaphocephaly due to premature closure of the sagittal suture was often (21%) seen in the severely affected MPS I (6 patients) and MPS VI (4 patients). In 13 % of all MPS patients, all sutures closed at an early age (pansynostosis), with normal head shape in two patients. Pansynostosis can easily be overlooked in these children, as their small head size can be interpreted as normal because they often have a small stature. In MPS patients in whom growth stagnation of the head occurs (example in Supplemental Fig. 1), further investigation is warranted, regardless of whether this is in line with their body length growth.

In MPS, evaluation of the consequences of craniosynostosis is complicated because increased ICP in this condition is often multifactorial. Hydrocephalus in MPS arises from the accumulation of GAGs in cells of the brain (ventricles, arachnoid villi), in supporting structures (meninges or spinal column), or results from venous hypertension related to the flow-limiting morphological changes in the skull base (Fig. 3c) and craniocervical junction (Alden et al. 2017; Dalla Corte et al. 2017). Moreover, the detection of clinical symptoms of raised ICP, such as visual decline or headache and nausea, can be difficult to detect, especially in the cognitively impaired patients.

Another pitfall in the evaluation of the consequences of craniosynostosis in MPS is the assessment of increased ICP in these disorders. Papilledema is not always present in MPS patients with increased ICP (for example, MPS VI patients, patient no. 3), while, vice versa, it can be present in patients with normal ICP, as a result of GAG accumulation in the sclera or optic nerve (Beck and Cole 1984; Collins et al. 1990). Expansion of the ventricles in response to increased ICP is often not present in MPS patients, as the ventricles are stiff due to the GAG accumulation, while enlarged ventricles can be present without raised ICP in the neuronopathic MPS patients due to brain atrophy (Alden et al. 2017). Thus, in the case of high clinical suspicion of increased ICP in MPS patients, thorough examination using different diagnostic modalities (including lumbar punction and/or 24-h ICP monitoring) should be carried out before dismissing this diagnosis.

When hydrocephalus and craniosynostosis occur in the same MPS patient, this can result in severely elevated ICP, since expansion of the skull in response to increase in pressure cannot take place. This is illustrated by the examples in our studied cohort. Patient 6 with MPS II did not have craniosynostosis and his skull could, therefore, expand to + 4SD in response to the occurring hydrocephalus, resulting in near-normal ICP. In contrast, patient 3 with MPS VI, in whom all sutures closed at a young age leading to skull growth stagnation, high ICP was found, potentially as a result of the combination of a CSF drainage problem and craniosynostosis.

In our patient cohort, craniosynostosis resulting in increased ICP also occurred in the non-neuronopathic MPS VI patients. This is demonstrated in the adult MPS VI patient in Fig. 3c, where the indentations of the brain in the skull, observed upon autopsy, indicate raised ICP earlier in life. In non-neuronopathic MPS patients, extensive GAG accumulation in the brain is not observed, and neurocognitive developmental is usually described as normal (Neufeld and Muenzer 2001; Valayannopoulos et al. 2010). Interestingly, we previously described mild cognitive impairment in three MPS VI patients (Ebbink et al. 2016). In this study, it is shown that two of these patients had pansynostosis and one had closure of two sutures before the age of 6 years (patient nos. 1, 5, and 6). Whether craniosynostosis indeed contributed to the cognitive disturbances in these patients remains to be determined by studying larger numbers of non-neuronopathic patients.

In other craniosynostosis syndromes such as Apert and Pfeiffer syndromes, suture closure occurs in utero, resulting in increased ICP very early in life (Mathijssen et al. 1999; Lajeunie et al. 2006). In these cases, guidelines for treatment in the form of surgical cranial vault expansion are clear (Mathijssen 2015). In MPS, suture closure seems to occur in early childhood; thus, the clinical consequences are likely to be less severe. Increased ICP in MPS can be multifactorial; treatment decisions should, thus, be made for each case individually, taking into account all aspects of the disorder. In the non-neuronopathic MPS patients, surgical cranial vault expansion might be an option in early childhood. In the neuronopathic patients, placement of a VPS to decrease ICP may be the treatment of choice since ongoing neurocognitive decline due to intracerebral GAG accumulation is to be expected and surgery for craniosynostosis imposes a large burden on the child.

In order to prevent complications of craniosynostosis, we recommend to monitor both skull growth by measuring head circumferences and to perform radiographs of the skull yearly in both neuronopathic and non-neuronopathic MPS patients until at least the age of 6 years.

## Conclusion

Craniosynostosis occurs in the majority of mucopolysaccharidosis (MPS) patients. Since the clinical consequences can be severe and surgical intervention is possible, skull growth and signs and symptoms of increased intracranial pressure (ICP) should be monitored in both neuronopathic and non-neuronopathic patients with MPS.

## Electronic supplementary material

Below is the link to the electronic supplementary material.

Supplemental Fig. 1 Example of early pansynostosis in an MPS VI patient. Patient no. 1, MPS VI, 9 years old. **a** Photographs show the distinct facial features and the normal shape of the skull. **b** X-skull at age 6 years of age shows closure of all sutures. **c** The growth curve shows stagnation of the skull growth (from − 1SD to − 2SD at around the age of 6 years). Decline in height (0SD to − 8.8SD) from the age of 1.5 years till 17 years old. (PNG 2370 kb)

High resolution image (TIF 19868 kb)

## References

[CR1] Alden TD, Amartino H, Dalla Corte A, Lampe C, Harmatz PR, Vedolin L (2017). Surgical management of neurological manifestations of mucopolysaccharidosis disorders. Mol Genet Metab.

[CR2] Beck M, Cole G (1984). Disc oedema in association with Hunter’s syndrome: ocular histopathological findings. Br J Ophthalmol.

[CR3] Bhattacharya K, Balasubramaniam S, Choy YS (2014). Overcoming the barriers to diagnosis of Morquio A syndrome. Orphanet J Rare Dis.

[CR4] Boulet SL, Rasmussen SA, Honein MA (2008). A population-based study of craniosynostosis in metropolitan Atlanta, 1989–2003. Am J Med Genet A.

[CR5] Brisman JL, Niimi Y, Berenstein A (2004). Sinus pericranii involving the torcular sinus in a patient with Hunter’s syndrome and trigonocephaly: case report and review of the literature. Neurosurgery.

[CR6] Cohen MM (1988). Craniosynostosis update 1987. Am J Med Genet Suppl.

[CR7] Cohen MM (1993). Sutural biology and the correlates of craniosynostosis. Am J Med Genet.

[CR8] Collins ML, Traboulsi EI, Maumenee IH (1990). Optic nerve head swelling and optic atrophy in the systemic mucopolysaccharidoses. Ophthalmology.

[CR9] Dalla Corte A, de Souza CFM, Anés M, Giugliani R (2017). Hydrocephalus and mucopolysaccharidoses: what do we know and what do we not know?. Childs Nerv Syst.

[CR10] de Jong T, Bannink N, Bredero-Boelhouwer HH, et al (2010) Long-term functional outcome in 167 patients with syndromic craniosynostosis; defining a syndrome-specific risk profile. J Plast Reconstr Aesthet Surg 63:1635–1641 10.1016/j.bjps.2009.10.029 19913472

[CR11] Ebbink BJ, Brands MM, van den Hout JM (2016). Long-term cognitive follow-up in children treated for Maroteaux–Lamy syndrome. J Inherit Metab Dis.

[CR12] Foo R, Whitaker LA, Bartlett SP (2010). Normocephalic pancraniosynostosis resulting in late presentation of elevated intracranial pressures. Plast Reconstr Surg.

[CR13] French LR, Jackson IT, Melton LJ (1990). A population-based study of craniosynostosis. J Clin Epidemiol.

[CR14] Lajeunie E, Heuertz S, El Ghouzzi V (2006). Mutation screening in patients with syndromic craniosynostoses indicates that a limited number of recurrent FGFR2 mutations accounts for severe forms of Pfeiffer syndrome. Eur J Hum Genet.

[CR15] Manara R, Priante E, Grimaldi M (2011). Brain and spine MRI features of hunter disease: frequency, natural evolution and response to therapy. J Inherit Metab Dis.

[CR16] Mathijssen IM (2015). Guideline for care of patients with the diagnoses of craniosynostosis: working group on craniosynostosis. J Craniofac Surg.

[CR17] Mathijssen IM, van Splunder J, Vermeij-Keers C (1999). Tracing craniosynostosis to its developmental stage through bone center displacement. J Craniofac Genet Dev Biol.

[CR18] Moosa S, Wollnik B (2016). Altered FGF signalling in congenital craniofacial and skeletal disorders. Semin Cell Dev Biol.

[CR19] Neufeld EF, Muenzer J, Scriver CR, Beaudet AL, Sly WS, Valle D (2001). The mucopolysaccharidoses. The metabolic bases of inherited disease.

[CR20] Persing JA, Jane JA, Shaffrey M (1989). Virchow and the pathogenesis of craniosynostosis: a translation of his original work. Plast Reconstr Surg.

[CR21] Ryoo HG, Kim SK, Cheon JE, Lee JY, Wang KC, Phi JH (2014). Slit ventricle syndrome and early-onset secondary craniosynostosis in an infant. Am J Case Rep.

[CR22] Sadashiva N, Bindu PS, Santosh V, Devi BI, Shukla D (2015). Mucopolysaccharidosis type I with craniosynostosis. Neurol India.

[CR23] Shapiro EG, Jones SA, Escolar ML (2017). Developmental and behavioral aspects of mucopolysaccharidoses with brain manifestations—neurological signs and symptoms. Mol Genet Metab.

[CR24] Shuper A, Merlob P, Grunebaum M, Reisner SH (1985). The incidence of isolated craniosynostosis in the newborn infant. Am J Dis Child.

[CR25] Singer S, Bower C, Southall P, Goldblatt J (1999). Craniosynostosis in Western Australia, 1980–1994: a population-based study. Am J Med Genet.

[CR26] Speltz ML, Kapp-Simon KA, Cunningham M, Marsh J, Dawson G (2004). Single-suture craniosynostosis: a review of neurobehavioral research and theory. J Pediatr Psychol.

[CR27] Tahiri Y, Bartlett SP, Gilardino MS (2017). Evidence-based medicine: nonsyndromic craniosynostosis. Plast Reconstr Surg.

[CR28] Taylor HR, Hollows FC, Hopwood JJ, Robertson EF (1978). Report of a mucopolysaccharidosis occurring in Australian aborigines. J Med Genet.

[CR29] Twigg SR, Wilkie AO (2015). New insights into craniofacial malformations. Hum Mol Genet.

[CR30] Valayannopoulos V, Nicely H, Harmatz P, Turbeville S (2010). Mucopolysaccharidosis VI. Orphanet J Rare Dis.

[CR31] Vu HL, Panchal J, Parker EE, Levine NS, Francel P (2001). The timing of physiologic closure of the metopic suture: a review of 159 patients using reconstructed 3D CT scans of the craniofacial region. J Craniofac Surg.

[CR32] Ziyadeh J, Le Merrer M, Robert M, Arnaud E, Valayannopoulos V, Di Rocco F (2013). Mucopolysaccharidosis type I and craniosynostosis. Acta Neurochir (Wien).

