# Supplementary figures and images for: Craniosynostosis affects the majority of mucopolysaccharidosis patients and can contribute to increased intracranial pressure
Source: J Inherit Metab Dis. 2018 Aug 6;41(6):1247–58. doi: 10.1007/s10545-018-0212-1 (PMC6326980; doi:10.1007/s10545-018-0212-1)

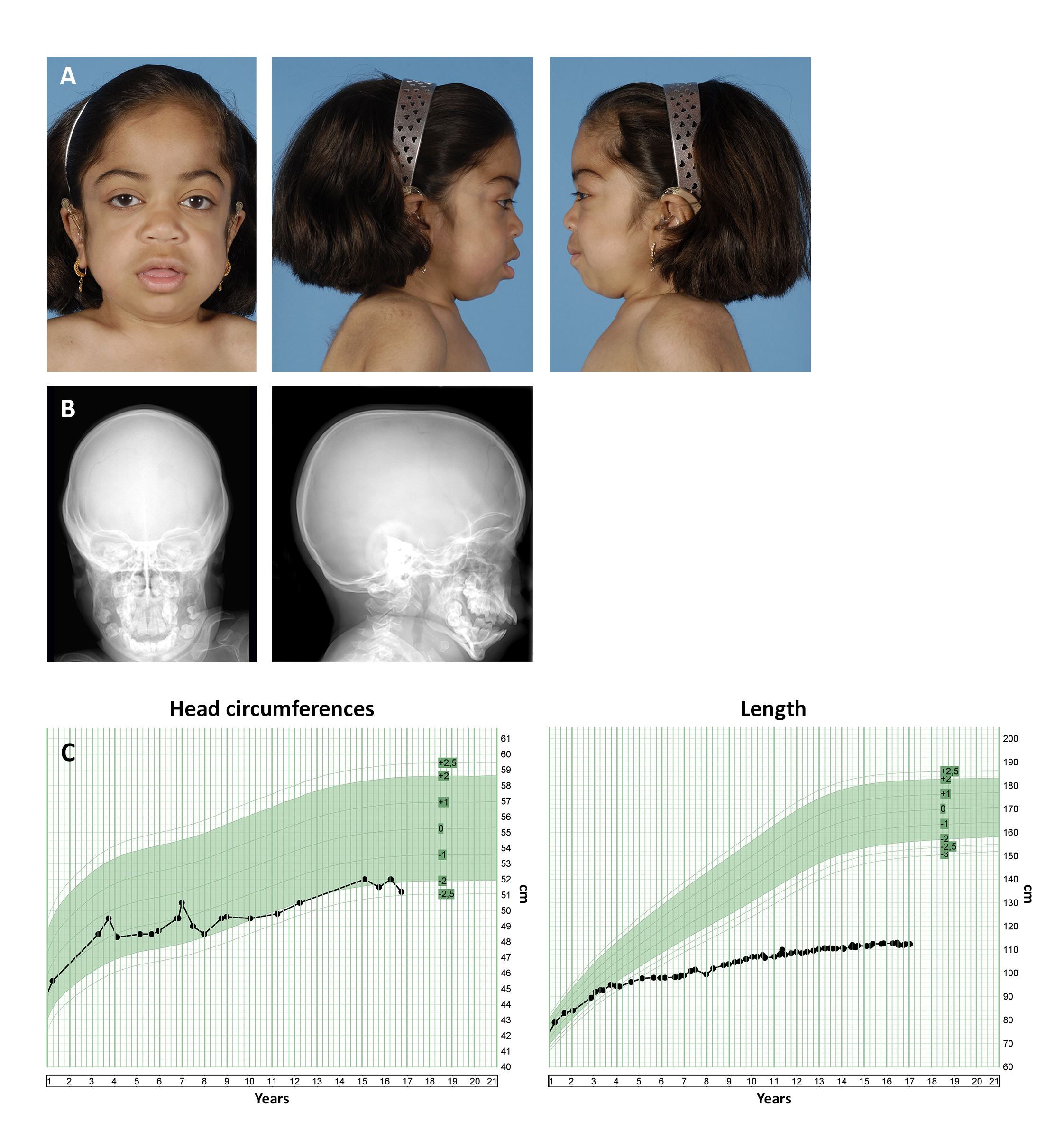

Supplement: Supplementary file 1 — Example of early pansynostosis in an MPS VI patient. Patient no. 1, MPS VI, 9 years old. a Photographs show the distinct facial features and the normal shape of the skull. b X-skull at age 6 years of age shows closure of all sutures. c The growth curve shows stagnation of the skull growth (from − 1SD to − 2SD at around the age of 6 years). Decline in height (0SD to − 8.8SD) from the age of 1.5 years till 17 years old. (PNG 2370 kb) [file 10545_2018_212_Fig4_ESM.png]

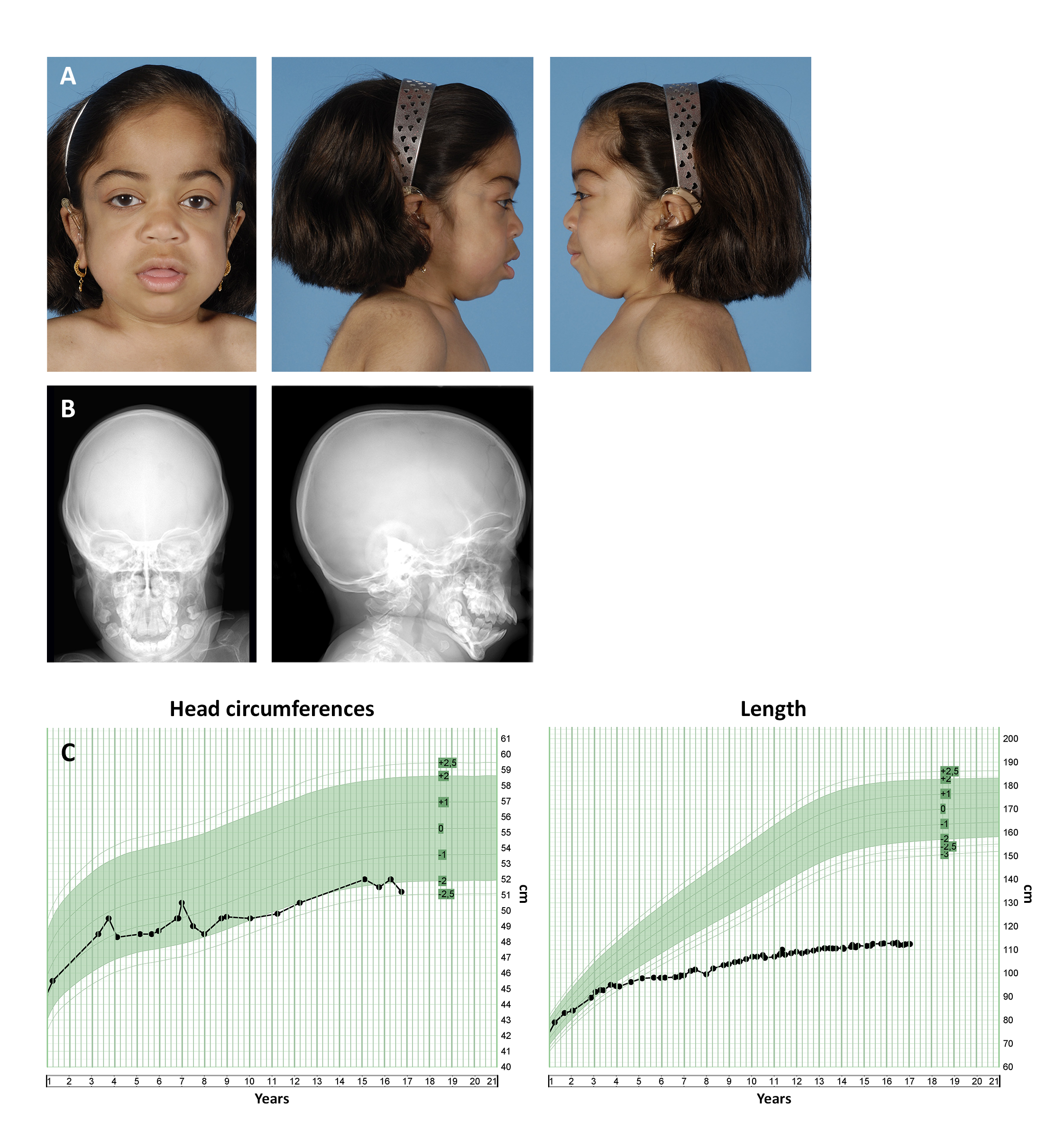

Supplement: Supplementary file 2 — High resolution image (TIF 19868 kb) [file 10545_2018_212_MOESM1_ESM.tif]
